# Acute Pancreatitis Secondary to Use of Appetite Suppressant: Garcinia cambogia

**DOI:** 10.7759/cureus.4676

**Published:** 2019-05-16

**Authors:** Umair Iqbal, Hafsa Anwar, Hafiz Umair Siddiqui, Asif Mehmood

**Affiliations:** 1 Internal Medicine, Geisinger Commonwealth School of Medicine, Danville, USA; 2 Internal Medicine, Jinnah Sindh Medical University, Karachi, PAK; 3 Surgery, Miller Family Heart and Vascular Institute, Cleveland Clinic, Cleveland, USA; 4 Internal Medicine, Geisinger Medical Center, Danville, USA

**Keywords:** garcinia cambogia, acute pancreatitis, appetite suppressants, weight loss medications

## Abstract

Due to the global epidemic of obesity, weight loss and appetite suppressant herbal products are quite popular. As these medications are not United States Food and Drug Administration-approved and are regulated as dietary supplements, little evidence exists regarding their safety. This case discusses an 82-year-old man with the past medical history of obesity who presented to the emergency department with abdominal pain in the epigastric region. His serum lipase was elevated, and an abdominal computed tomography revealed acute pancreatitis (AP). He reported two episodes of AP in the past. He denied any alcohol use and reported no recent changes in his medications. He reported taking *Garcinia cambogia* (GC) recently as an appetite suppressant. Due to prior cholecystectomy, no alcohol abuse, no recent changes in medications and recent use of GC, a likely etiology of AP was thought to be secondary to the use of GC. He was treated with bowel rest and intravenous fluid hydration with significant improvement in his symptoms. He was advised to avoid GC in the future. Clinicians should be vigilant in evaluating their patients with AP and should get a meticulous history regarding their use of over-the-counter medications and herbal products.

## Introduction

An obesity epidemic is on the rise globally, causing weight loss medications to gain popularity; however, they carry a significant risk of adverse events [1-2]. Herbal supplements are not United States Food and Drug Administration-approved and are regulated as dietary products. Acute pancreatitis (AP) is one of the more common causes of hospitalization [3]. Common etiologies of AP include gallstones, alcohol, and medications [3]. *Garcinia cambogia* (GC) has been shown to be effective as an appetite suppressant. Hydroxy citric acid is an active ingredient of this herbal product, which competitively inhibits adenosine triphosphate citrate lyase and prevents the conversion of citrate to oxaloacetate and acetyl coenzyme A [4]. This results in a reduction in fatty acid synthesis. Several adverse effects have been reported secondary to the use of GC [5-12]. Herein, we discuss the case of an elderly man who presented to the emergency department with abdominal pain and was found to have acute pancreatitis (AP) likely secondary to the consumption of GC.

## Case presentation

An 82-year-old man with a past medical history significant for obesity, AP secondary to cholelithiasis, prior cholecystectomy, hypertension, hyperlipidemia, diabetes mellitus, pulmonary embolism, and nonischemic cardiomyopathy presented to the emergency department with concerns of abdominal discomfort for two days. The patient reported discomfort in the epigastric region, which was non-radiating. He reported nausea but no vomiting. He reported at least two bouts of AP in the past. The patient denied any fevers, chills, lightheadedness, headaches, chest pain, shortness of breath, hematochezia, numbness, or melena. His medications included aspirin, apixaban, atorvastatin, metformin, metoprolol, spironolactone, and ramipril.

**Figure 1:**
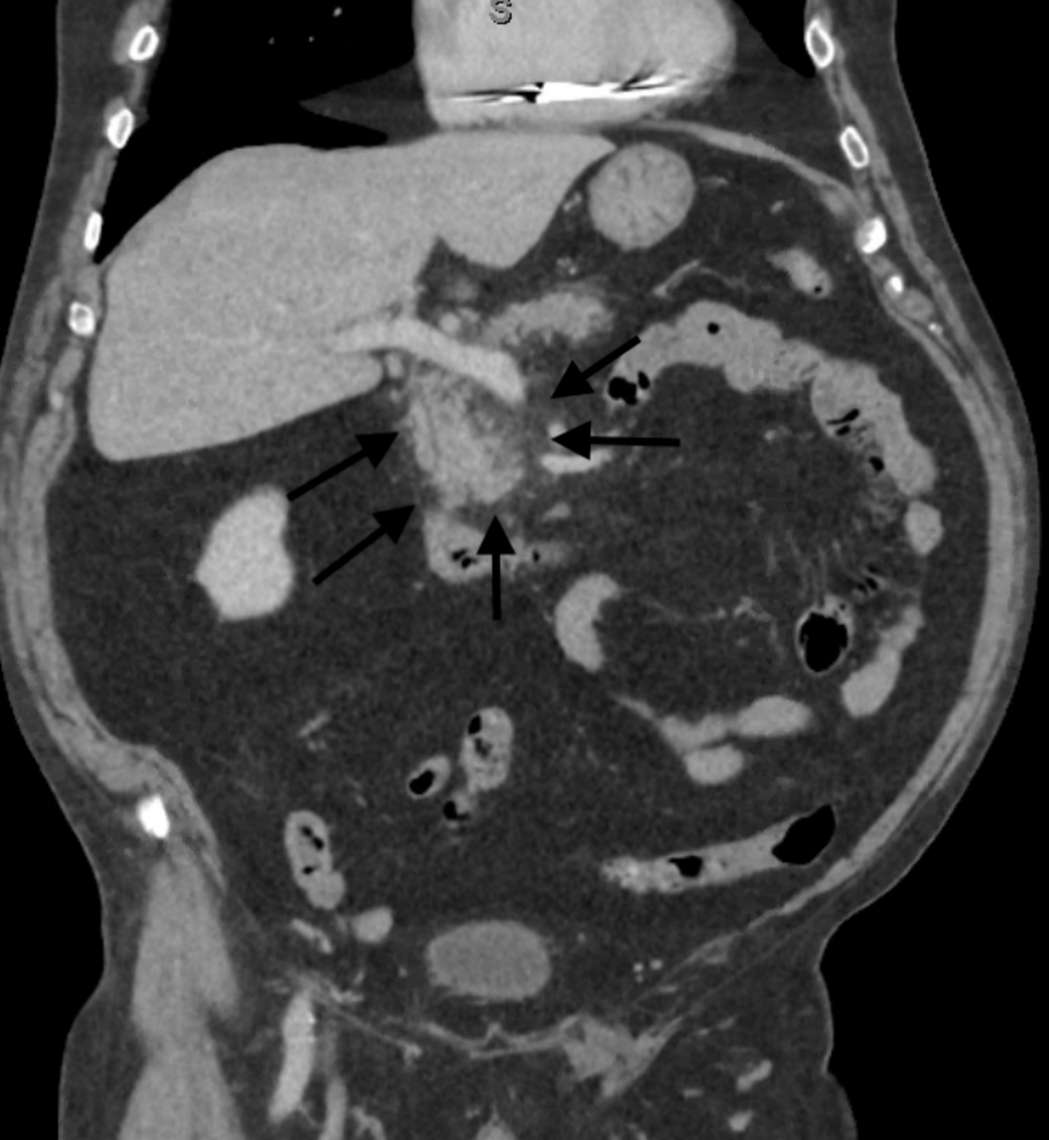
Coronal view of CT scan of abdomen pelvis in porto-venous phase showing fat stranding (black arrows) surrounding the head of the pancreas, compatible with acute pancreatitis CT, computed tomography

The patient reported taking GC as an appetite suppressant. On physical exam, tenderness was noted in the epigastric area, and bowel sounds were positive. His serum lipase was elevated to >600 U/L (reference range, 16 to 63 U/L), white blood cell count was 11.7 K/µL (reference range, 4.5 to 11 K/µL), hemoglobin was 13.4 g/dL (reference range, 13.5 to 17.5 g/dL), bilirubin was 0.5 mg/dL (reference range,0.1 mg/dl to 1.2 mg/dl), alkaline phosphatase was 66 IU/L (reference range 44 to 147 IU/L), alanine aminotransferase was 12 U/L (reference range, 10 U/L to 40 U/L), aspartate aminotransferase was 17 U/L (reference range, 10 to 40 U/L), and lactic acid was 0.9 mmol/L (reference range, 0.5 to 2.0 mmol/L). A computed tomography scan of the abdomen revealed fat stranding around his pancreas, which was concerning for AP (Figure 1).

The patient denied any use of alcohol. Triglycerides levels were 146 mg/dL (reference range, <200 mg/dL). He was hospitalized and treated with adequate intravenous hydration, nothing by mouth, with remarkable improvement in his symptoms within 24 hours of presentation. He tolerated regular oral diet on discharge from the hospital. Due to his relatively recent consumption of GC and no recent changes in any other medications, a likely etiology of AP was thought to be secondary to the use of this herbal product. His care team recommended he avoid the use of GC in the future.

## Discussion

The consumption of dietary and herbal supplements is rising worldwide [2]. GC is a fruit found in Southeast Asia and West Africa. GC has been marketed as a weight loss supplement, and several studies have been conducted to evaluate its efficacy in animals and humans. GC has been reported to increase the release of serotonin in the brain and can lead to suppression of appetite [13]. It has also been shown to reduce carbohydrate metabolism by inhibition of pancreatic alpha-amylase and intestinal alpha-glucosidase [14]. However, a meta-analysis of nine randomized controlled trials revealed a very small reduction in weight using GC compared to placebo [15]. The study concluded that GC might cause short-term weight loss effects, but the clinical significance of the small effects remained unclear and requires future larger prospective trials.

Multiple cases have been reported in the past regarding AP secondary to the use of GC. Grigos et al. reported an elderly woman who presented with abdominal pain and was found to have AP secondary to use of GC [5]. Similarly, Bystrak et al. reported a case of diabetic ketoacidosis and AP secondary to regular use of GC in a middle-aged woman with diabetes [6]. The pathogenesis regarding how GC increases the risk of AP is unclear. Reactive oxygen species plays a pivotal role in the pathogenesis of AP. GC increases lipid peroxidation and upregulates the superoxide dismutase and glutathione peroxidase messenger Ribonucleic acid (RNA) expression, which might be a secondary compensatory response due to increased oxidative stress from use of GC [16]. Lipid peroxidation also increases oxidative stress and might increase the risk of AP in patients consuming GC [17]. GC can also cause other serious adverse events including hepatoxicity [7-8]. Lunsford et al. reported a case of fulminant hepatic failure secondary to ingestion of GC in a young man who eventually required orthotopic liver transplantation [7]. Corey et al. also reported a case of acute liver failure secondary to use of GC requiring liver transplantation [8]. There have also been reports of acute necrotizing eosinophilic myocarditis, rhabdomyolysis, serotonin toxicity, and nephropathy secondary to use GC [9-12].

## Conclusions

Consumption of GC increases the risk of AP. Given its only small short-term effect on weight loss, GC should be avoided until larger prospective trials are done to evaluate its efficacy and safety in the general population. Physicians should obtain a thorough clinical history regarding the use of herbal supplements in patients with AP, as patients usually underreport their use of these products.
